# Summary of the COVID-19 epidemic and estimating the effects of emergency responses in China

**DOI:** 10.1038/s41598-020-80201-8

**Published:** 2021-01-12

**Authors:** Junwen Tao, Yue Ma, Caiying Luo, Jiaqi Huang, Tao Zhang, Fei Yin

**Affiliations:** West China School of Public Health and West China Fourth Hospital, Sichuan University, Chengdu, China

**Keywords:** Infectious diseases, Public health

## Abstract

Coronavirus disease-2019 (COVID-19) pandemic has affected millions of people since December 2019. Summarizing the development of COVID-19 and assessing the effects of control measures are very critical to China and other countries. A logistic growth curve model was employed to compare the development of COVID-19 before and after the emergency response took effect. We found that the number of confirmed cases peaked 9–14 days after the first detection of an imported case, but there was a peak lag in the province where the outbreak was concentrated. Results of the growth curves indicated that the fitted cumulative confirmed cases were close to the actual observed cases, and the *R*^2^ of all models was above 0.95. The average growth rate decreased by 44.42% nationally and by 32.5% outside Hubei Province. The average growth rate in the 12 high-risk areas decreased by 29.9%. The average growth rate of cumulative confirmed cases decreased by approximately 50% after the emergency response. Areas with frequent population migration have a high risk of outbreak. The emergency response taken by the Chinese government was able to effectively control the COVID-19 outbreak. Our study provides references for other countries and regions to control the COVID-19 outbreak.

## Introduction

On December 31, 2019, China notified the World Health Organization (WHO) of unknown pneumonia cases in Wuhan, Hubei province^1^. This pneumonia came with persistent fever, cough, and dyspnea^2^ and was then named Coronavirus Disease 2019 (COVID-19). The disease spread rapidly from Hubei province to other provinces in China within 2 weeks^3,4^. By November 14, 2020, a total of 92,409 confirmed cases and 4749 deaths had been reported in China, of which less than seventeen percent of the cases and less than four percent of the deaths occurred outside Hubei province. Since 13 January 2020, first Thailand^5^, then more than 200 countries, including Japan, Korea^6^, the United States^7^, and the United Kingdom^8^, have reported imported COVID-19 cases. Due to the speed and scale of transmission, the WHO described COVID-19 as a pandemic on 12 March 2020, officially declaring that COVID-19 entered the global epidemic phase.

Beginning 15 January 2020, the Chinese government launched an emergency response at all levels. On the one hand, in the epicenter of the outbreak, Hubei province implemented traffic control. On the other hand, the whole nation was required to wear masks and to avoid going out and having close contact with other people to reduce the exposure to susceptible people. As the earliest occurrence area, Hubei province has been through the process of case accumulation—outbreak detection—isolation and control. The rest of China has been through a complete process of case imports—detected transmission—isolation and control. Besides, as winter comes, the second wave of COVID-19 becomes one of the most important concerns of China and other countries. Those who have managed to take the COVID-19 epidemic under control are now threatened by the risk from imported cases while those who failed to flatten the epidemic curves are accumulating active cases continuously. Therefore, summarizing the COVID-19 development in Hubei province and other regions of China can help us to explore the epidemic characteristics of COVID-19 and provide a reference for other countries to assess the stages of the COVID-19 epidemic.

The course of COVID-19 includes incubation, disease, and recovery or death^2,9^. This course is characterized at the population level, as the number of cumulative confirmed cases experience a period of delay before exponential growth, then present a period of maximum increasing density, and finally enter a stable stage. The entire process presents an s-shaped development trend. A logistic growth curve model^10^ is often used to describe such ecological processes^11,12^. Both the average growth rate and the maximum value of the growth curve have clear epidemiological significance and are of great reference value in the field of public health. Therefore, this study used the logistic growth curve model to evaluate the effects of emergency responses before and after implementation in two situations. One is in the epicenter, Hubei Province, in which numerical local transmissions have already occurred when the COVID-19 cases were firstly reported. The other is the regions with large immigration from the epicenter, mainly threatened by the importing risk. In addition, this study would extract historical data to simulate a short-term dynamic prediction and discussed the application of the growth curve model in the assessment of COVID-19 to provide a reference for China and other countries.

## Results

### General characteristics of COVID-19 in China

**Figure 1:**
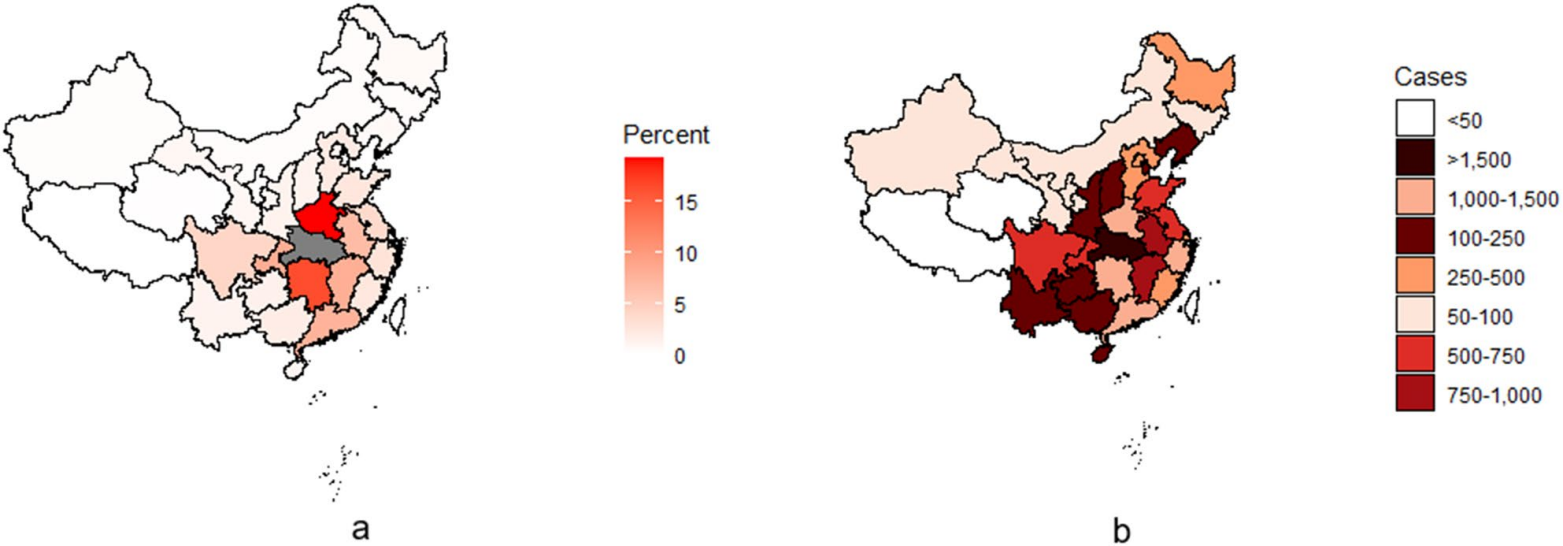
(**a**) Percentage of the migration population moving from Hubei province to other provinces on 22 January 2020. (**b**) The cumulative confirmed COVID-19 cases in Chinese provinces from 22 January to 4 March 2020.

Wuhan, Hubei province shut down outward traffic beginning 23 January 2020, followed by the rest of Hubei province. To find high-risk areas caused by imported cases, we drew a heatmap of the migration out of Hubei on 22 January 2020 (Fig. 1a), which indicated that people mainly migrated to Henan, Hunan, Chongqing, Jiangxi, Guangdong, Anhui, Sichuan, Jiangsu, Zhejiang, Beijing, and Shanghai. A heatmap of the cumulative confirmed cases in Chinese provinces from 22 January to 4 March 2020 highlights similar provinces (Fig. 1b). Hubei province was the location of the concentrated COVID-19 outbreak, followed by its neighbors (Henan, Anhui, Jiangxi, Hunan, and Chongqing) and some economically developed and densely populated provinces (Guangdong, Zhejiang, Jiangsu, Shandong, Sichuan, Shanghai, and Beijing). Thus, Sichuan, Guangdong, Beijing, Shandong, Chongqing, Zhejiang, Jiangxi, Anhui, Jiangsu, Hunan, Shanghai, and Henan were selected as high-risk areas with imported cases for further analysis. In addition, since over 80% of confirmed cases were reported in Hubei province (Table 1: 81,047 cases were reported in China, in which 67,990 cases were reported in Hubei province), the epidemic characteristics in the rest part of China may be masked by that in Hubei province. Therefore, we also analyzed the national data excluding Hubei province to present the epidemic development in other provinces.

**Table 1:** Peak number of confirmed COVID-19 cases, corresponding peak date, and cumulative confirmed cases in China, Hubei province, and 12 high-risk provinces from 22 January to 4 March 2020.

Area	Peak confirmed cases	Peak date	Cumulative cases
China	3893	2/4/2020	81,047
Hubei	3156	2/4/2020	67,990
China except for Hubei	890	2/3/2020	13,057
Sichuan	36	1/30/2020	538
Guangdong	127	1/31/2020	1325
Beijing	32	2/2/2020	411
Shandong	45	2/5/2020	757
Chongqing	38	2/2/2020	571
Zhejiang	132	1/29/2020	1209
Jiangxi	85	2/3/2020	937
Anhui	72	2/3/2020	991
Jiangsu	37	2/3/2020	631
Hunan	74	2/1/2020	1017
Shanghai	27	1/30/2020	329
Henan	109	2/3/2020	1273

**Figure 2:**
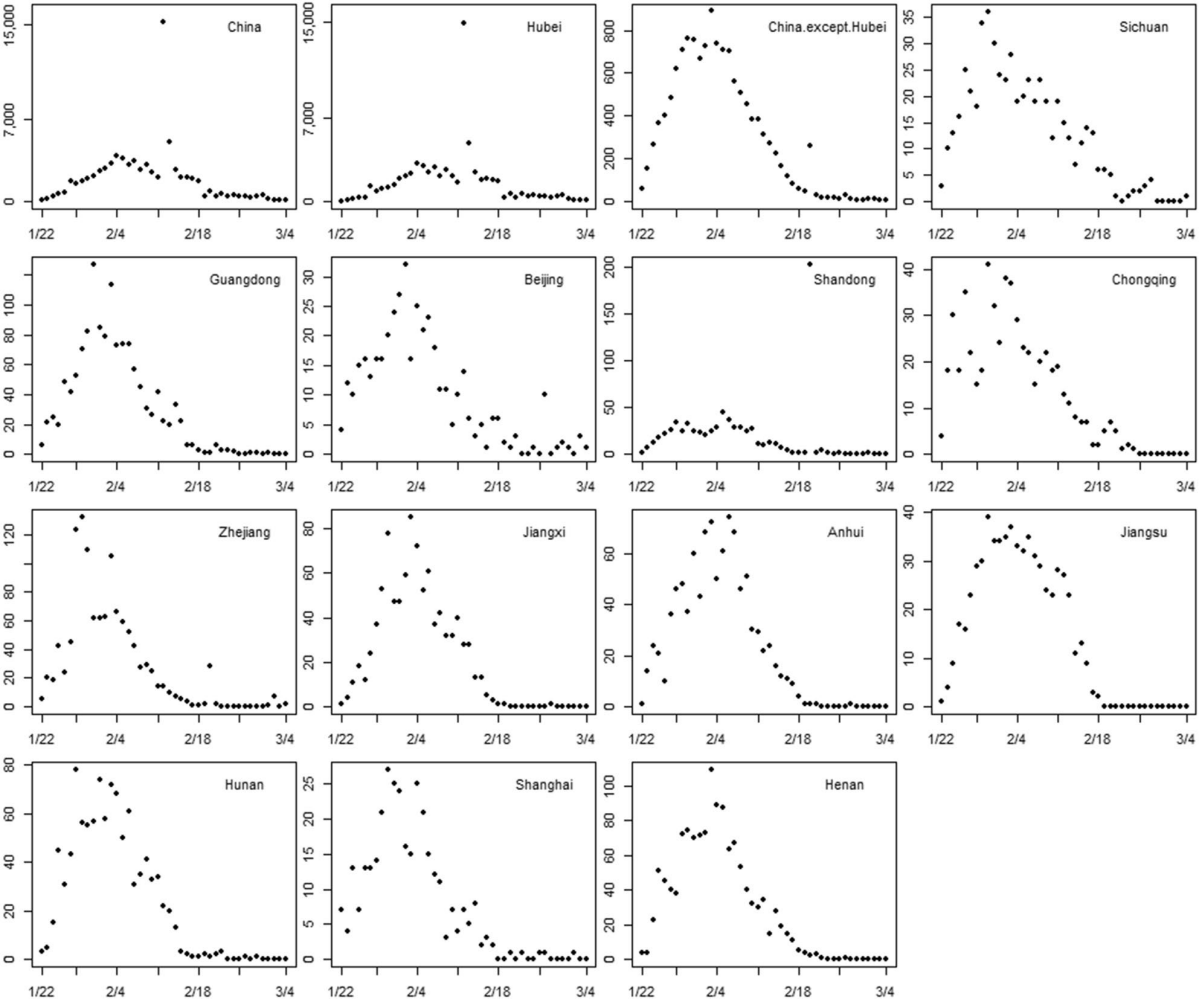
The time series of confirmed COVID-19 cases in China, Hubei province, and 12 high-risk provinces from 22 January to 4 March 2020.

According to the time series of the confirmed COVID-19 cases (except outliers) in China and 12 high-risk provinces, we summarized the peak confirmed cases, the corresponding peak date, and the cumulative number of confirmed cases (Table 1). Figure 2 shows the time series of confirmed COVID-19 cases in the identified provinces. The confirmed COVID-19 cases in Hubei province and nationwide showed a rapid increase before February 4, followed by a decline, and gradually stabilized after February 18, 2020. In high-risk provinces with imported cases, the peak of confirmed cases was around 30 January 2020 in Sichuan, Guangdong, Zhejiang, and Shanghai, and around 2 February 2020 in Beijing, Chongqing, Jiangxi, Anhui, Jiangsu, Hunan, and Henan.

Two outliers occurred in China and Hubei province on February 12 and 13, as the National Health Commission of the PRC revised the definition of COVID-19 confirmed cases in Hubei province on February 12, adding “clinical case” to “confirmed case,” and left the other provinces unchanged^13^. Another outlier was found in Shandong Province on February 20, corresponding to an outbreak at a prison with 200 confirmed cases^14^. The overall trend of confirmed cases in the other provinces increased first and then decreased.

### Impact evaluation of emergency response

**Figure 3:**
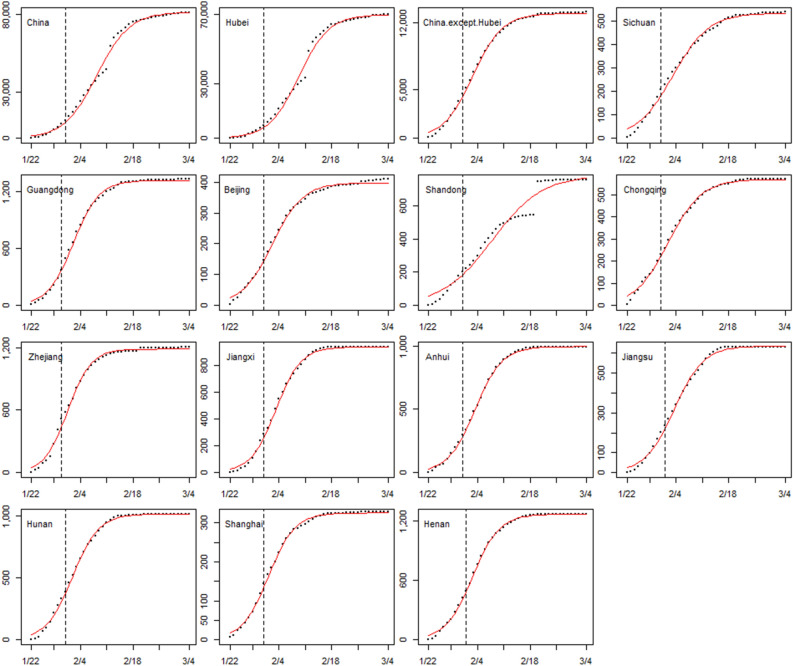
The logistic growth curves of China, Hubei province, and 12 high-risk provinces before and after the emergency responses. Black points representing observed values, red lines representing fitted growth curves, and black dash lines representing two different periods’ cut-off points.

We fitted the growth curves at two different periods to assess the impact of the emergency response implemented in each province. Figure 3 shows the growth curves of each area. The coefficients of the logistic growth curve models in two periods are referred to the Supplementary Tables S2 and S3. The fitted cumulative confirmed cases were close to the actual observed cases, and the *R*^2^ of all models was above 0.95.

**Figure 4:**
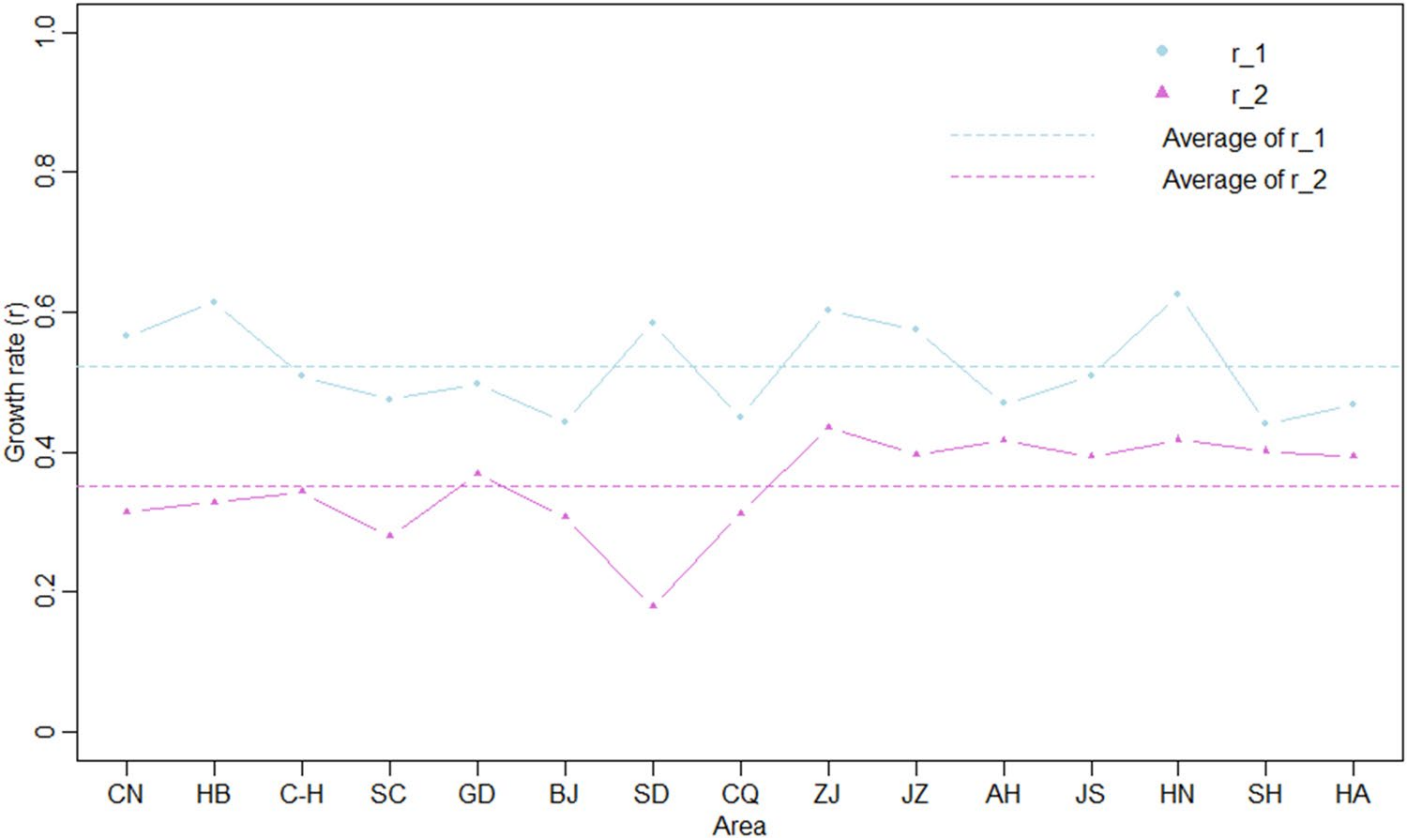
The comparison of the average growth rates before and after the emergency responses in China, Hubei province, and 12 high-risk provinces.

The average growth rates of the two periods in China, Hubei province, and 12 high-risk provinces are presented in Table 2 and Fig. 4. The average growth rate decreased by 44.4% nationally and by 32.5% outside Hubei province. The average growth rate in each province decreased significantly after the emergency response. The average growth rate in the 12 high-risk areas decreased by 29.9%, which was lower than that outside Hubei province. Before the emergency response, the provinces with the highest average growth rates were ranked from highest to lowest as follows: Hunan, Hubei, Zhejiang, Shandong, Jiangxi, Jiangsu, Guangdong, Sichuan, Anhui, Henan, Chongqing, Beijing, and Shanghai. Hubei, Shandong, Zhejiang, Jiangxi, and Hunan had growth rates higher than the national average. After the emergency response, the average growth rate of each province from highest to lowest was Zhejiang, Hunan, Anhui, Shanghai, Jiangxi, Jiangsu, Hunan, Guangdong, Hubei, Chongqing, Beijing, Sichuan, and Shandong. The growth rates of Guangdong, Zhejiang, Jiangxi, Anhui, Jiangsu, Hunan, Shanghai, and Henan were higher than the national average.

**Table 2:** Comparison of the average growth rates before and after the emergency responses in China, Hubei province, and 12 high-risk provinces.

Area	r_1^a^	r_2^b^	Percentage decrease
China	0.565	0.314	0.444
Hubei	0.614	0.328	0.466
China except for Hubei	0.508	0.343	0.325
Sichuan	0.475	0.279	0.413
Guangdong	0.498	0.370	0.257
Beijing	0.443	0.308	0.305
Shandong	0.584	0.179	0.693
Chongqing	0.450	0.313	0.304
Zhejiang	0.603	0.435	0.279
Jiangxi	0.576	0.397	0.311
Anhui	0.469	0.417	0.111
Jiangsu	0.509	0.393	0.228
Hunan	0.625	0.418	0.331
Shanghai	0.440	0.402	0.086
Henan	0.468	0.393	0.160
Average of 12 high-risk areas	0.512	0.359	0.299

^a^r_1: Average growth rate before the emergency responses.

^b^r_2: Average growth rate after the emergency responses.

### Prediction capacity evaluation of logistic growth curve models

**Figure 5:**
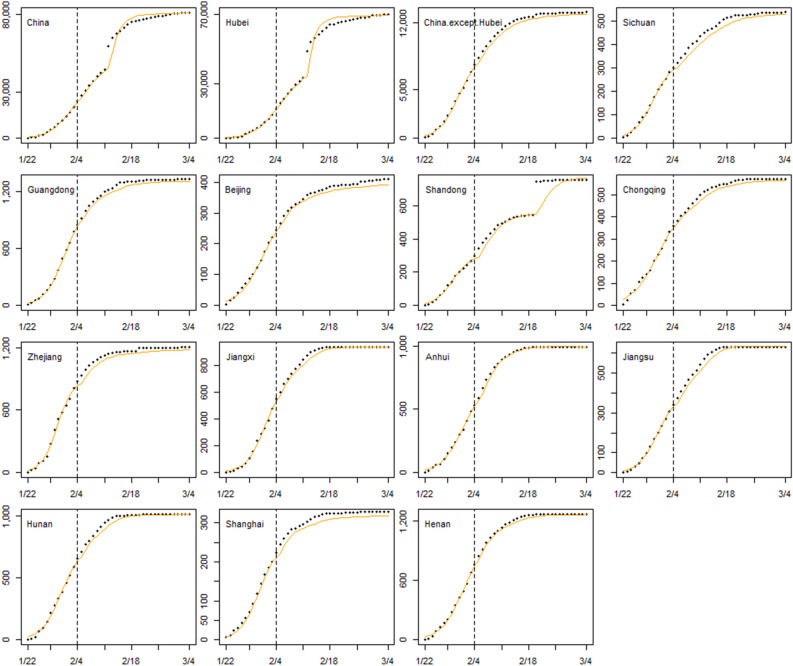
The 1-step dynamic prediction of the logistic growth curve model in China, Hubei province, and 12 high-risk provinces. Black points representing observed values and orange lines representing fitted growth curves.

We used cumulative confirmed case data, from January 22 to February 4, 2020, to simulate a short-term dynamic prediction. Table 3 shows the MAE and MAPE of the logistic growth curve model in each province. Figure 5 shows the 1-step dynamic prediction of the logistic growth curve model in China, Hubei province, and 12 high-risk provinces. The 1-step dynamic prediction outperformed the rest, with a MAPE of 1.16–5.45% in different areas. Except for the models for China, Hubei, and Shandong provinces, which were affected by the three outliers mentioned above, the models showed predictions close to the observations.

**Table 3:** MAE and MAPE of the logistic growth curve model in China, Hubei province, and 12 high-risk provinces.

Area	MAE			MAPE(%)		
	1 out-of-sample	3 out-of-sample	7 out-of-sample	1 out-of-sample	3 out-of-sample	7 out-of-sample
China	1322.3	2170.57	2285.19	3.54	5.02	13
Hubei	1392.29	2472.08	2290.85	4.05	6.28	14.04
China except for Hubei	1780.99	1781.32	1805.11	3	3.97	6.55
Sichuan	2.83	7.7	11.1	4.58	5.88	8.77
Guangdong	4.81	9.62	16.37	2.64	3.3	4.99
Beijing	2.41	3.39	3.77	3.43	3.97	5.78
Shandong	17.6	23.49	27.35	5.45	7.71	12.95
Chongqing	2.17	4.03	6.04	2.7	3.33	4.73
Zhejiang	5.24	13.98	17.22	3.4	4.27	6.52
Jiangxi	3.92	9.04	13.7	1.72	2.57	4.83
Anhui	3.59	9.33	15.49	1.16	1.82	4.3
Jiangsu	2.78	9.69	13.33	2.71	3.94	7.2
Hunan	4.15	10.34	14.66	2.03	2.86	5.07
Shanghai	1.75	3.13	4.28	4.48	5.28	7.5
Henan	4.69	8.73	14.2	1.68	2.35	4.28

## Discussion

COVID-19 has currently become one of the biggest threats to the human world^15,16^. As the country reported the COVID-19 outbreak firstly, China issued national emergency responses^17,18^, including cross-regional traffic control and suspending the operations of restaurants, entertainment, and cultural tourism areas, and has taken the epidemic under control in early March. This study used the logistic growth curve models to summarize the COVID-19 epidemic in the epicenter, Hubei province, and other 12 high-risk provinces in China before and after the emergency responses. Results showed the areas with larger migration from Hubei province, have suffered more severe epidemics of the COVID-19. Prompt emergency responses after the detection of imported cases greatly reduced the growth rate of the local epidemic. Also, in the early stage without adequate information for more detailed dynamic prediction models, the logistic growth curve model has good prediction accuracy in the short-term forecast.

Before the shutdown of the traffic leaving Wuhan, Hubei province, people from Hubei province mainly migrated to Henan, Hunan, Chongqing, Jiangxi, Guangdong, Anhui, Sichuan, Jiangsu, Zhejiang, Beijing, and Shanghai, which was consistent with provinces later had high incidences of COVID-19. It indicated that the people migration was related to the spread of the COVID-19 epidemic. This finding can be supported by other studies^19,20^. As a respiratory infectious disease, the number of transmission sources and susceptible population density directly affects the COVID-19 spread^21^. Blocking migration from severe outbreak areas would be of great importance to prevent the disease from spreading to other areas, especially during the early stages. Tian et al. found that the confirmed cases reported in lockdown cities decreased by 37% than those cities without lockdown in China^22^. In addition, Flaxman et al. estimated the effects of non-pharmaceutical interventions on COVID-19 in 11 European countries and found that lockdown had a large effect on controlling the epidemic^23^.

The peak outbreak occurred from February 1 to February 4, 2020, which could be related to the population migration and the incubation of COVID-19. As January 25 was the traditional Chinese New Year, most people were returning to their hometowns to reunite with their families. Therefore, the densified migration in the week before the traditional Chinese New Year led to the rapid spread of COVID-19. With the estimated 3–7 days incubation, each province experienced 9–14 days from the first detection of imported cases to the peak of confirmed cases, which was consistent with the sum of the migration peak and the incubation period. Therefore, 9–14 days after the detection of imported cases is the critical period for preventing further transmission. In this period, screening tests and the quarantine of COVID-19 patients should be carried out to find the infection source and protect susceptible populations. Notably, in the region with the most severe outbreak, Hubei province, the peak of confirmed cases was delayed, which is consistent with the findings of Sun et al.^24^. They found that delays between suspected infection and seeking care at a hospital were longer in Hubei province than in other provinces. This phenomenon may be attributed to the long accumulation of confirmed cases and inadequate testing capacity, suggested that more health resources are needed in such an area.

The logistic growth curves of cumulative COVID-19 cases before and after the implementation of emergency response in each study province showed an approximate 50% reduction in the average growth rate after the emergency response, similar to the result of Lai’s study. Lai et al. predicted the confirmed cases would have been 67-fold higher by 29 February 2020 without the emergency response in China^25^. As all the emergency responses were launched within 1 week after the first confirmed case, the reduction in the average growth rate suggested that rapid growth of the epidemic can be slowed by a timely emergency response after the early detection of imported cases within the critical period of 9–14 days.

The average growth rate in Zhejiang, Jiangsu, Anhui, Jiangxi, Hunan, Shanghai, and Henan provinces remained higher than the national average growth rate after the implementation of the emergency response. Among them, the economically developed provinces, and labor-exporting provinces with frequent population migration, such as Zhejiang, Hunan, and Anhui provinces, had the highest growth rates, indicating a high outbreak risk. Therefore, the control measures should be particularly strengthened to prevent COVID-19 outbreaks in these regions. Although the emergency response reduced the average growth rate, in the outbreak center, Hubei province, the peak in confirmed cases was delayed. This suggests that if the outbreak was not detected in time, the critical control period might pass, which would lead to a lag in the implementation of prevention and control measures in response to the outbreak. Therefore, for concentrated COVID-19 outbreak areas, the growth of the epidemic would not be easily controlled within the standard critical period of 9–14 days. The lagged peak of confirmed cases should be fully considered, and the duration of control measures should be extended for further development of the epidemic. And a study in the UK suggested that to avoid a rebound, the control measures should be maintained until a vaccine is available, which might be about 18 months^26^.

In the 1-step dynamic prediction of the cumulative confirmed COVID-19 cases in the early stage of the epidemic, the MAPE between the predicted and actual cumulative cases was 1.16–5.45%. Despite the increase due to the change in diagnostic criteria on February 13 in Hubei province, the values predicted by the logistic growth curve model were very close to the actual observed values. Thus, the logistic growth curve model can be used to assess the short-term development of COVID-19 and aid in the short-term adjustment of prevention and control measures, especially in the early stage of the epidemic.

There are several limitations to this study. As based on the existing surveillance data, the detection capacity of COVID-19 varies between different regions, which may lead to an underestimated occurrence at the early stage, and the outbreak reflected by the surveillance data may be delayed. Each region should consider local detection capacity when formulating prevention and control measures.

In conclusion, areas with frequent migration have a high risk of COVID-19 outbreak, so the prevention and control measures should be strengthened. Timely detection of imported cases and blocking migration from the epidemic areas are important for controlling the spread of COVID-19. The 9–14 days after the first detection of imported cases could be the critical period for epidemic prevention and control. In areas where the epidemic is severe, we need to consider the peak lag and extend prevention measures. The emergency responses launched in China efficiently reduced the spread and further development of the epidemic, which provides a reference for other countries and regions, especially facing a new wave coming with the winter. The logistic growth curve model can accurately evaluate and predict the short-term development of the COVID-19 epidemic.

## Methods

### Data sources

Confirmed COVID-19 case data were obtained from the *Chinese Center for Disease Control and Prevention*^27^. All cases were confirmed by laboratory and clinical diagnosis and met the definition of confirmed cases according to *the National Health Commission of China*^28^. Baidu is the most widely used search engine in China, and we extracted population migration data from the *Baidu Qianxi* to find areas with early imported cases^29^. Considering that in the early stages of the COVID-19 outbreak, the situation reports may have underreported cases while the national new daily case has been reduced to the level around one hundred to the beginning of the March and kept at a low level in further, we used confirmed cases from 22 January to 4 March 2020 to ensure the reliability of the data.

### Statistical analysis

This study used heatmaps to conduct a spatiotemporal distribution analysis of cumulative confirmed COVID-19 cases and population migration in China on a provincial level. The heatmaps were constructed with the cumulative confirmed cases and population migration data via the “rgdal” and “ggplot2” packages in R 3.6.3. We selected Hubei province as the concentrated outbreak area for analysis, and other provinces with early reported cases as representative provinces facing the risk of an outbreak.

The logistic growth curve is a statistical model used to simulate the growth of cells, animals, plants, or populations. In a finite population, the logistic growth curve presents s-shaped. The parameters of this model have clear epidemiological significance and are of great reference value in the field of public health. Therefore, this study used the logistic growth curve model to summarize the characteristics of the COVID-19 epidemic and to evaluate the effects of emergency responses in China. The formula for the model is as follows:

$${N}_{t}=\frac{{N}_{0}K}{{N}_{0}+\left(K-{N}_{0}\right){e}^{-rt}}$$

where N_t_ represents the cumulative confirmed COVID-19 cases at time t, N_0_ represents the cumulative confirmed cases at the initial time, K represents the maximum cumulative confirmed cases within the analysis period, and r is the average growth rate of the cumulative confirmed cases. The “SummarizeGrowth()” function was used to fit the growth curve model via the “growthcurver” package in R 3.6.3.

To evaluate the effects of the emergency response implemented in each province, we fitted the logistic growth curve models at two different periods, using an average incubation period of 7 days^24,28^ after the emergency response implemented date as the cut-off point (for details of the time period, see Supplementary Table S1). The first time period was used to assess the situation before the emergency response. The second time period, from the end of period one to 4 March 2020, was used to assess the situation after the emergency response had taken effect. The coefficient of determination (*R*^2^) was used to evaluate the goodness of fit. The average growth rates of periods one and two in each province were compared to evaluate the effects of the emergency response.

To simulate the short-term trend of the epidemic, we used the logistic growth curve model for dynamic prediction from 22 January to 4 March 2020^30^. The step lengths of the dynamic predictions were set as 1, 3, and 7 days, referred to as the 1, 3, or 7 out-of-sample prediction. In the 1 out-of-sample prediction, the cumulative confirmed cases from January 22 to February 4 were selected as the training set, and 1 day after, February 5, was selected as the test set. Then, the model was updated with actual observations from February 5, and the cumulative confirmed cases on February 6 were predicted by the updated model until all the predicted cumulative confirmed cases from February 5 to March 4 were obtained. The average absolute error (MAE) and average absolute percentage error (MAPE) were then calculated for each dynamic prediction with different step lengths to evaluate the short-term trend of the epidemic.

All statistical analyses were performed in R 3.6.3 using packages such as “growthcurver”, “rgdal” and “ggplot2”.

## Supplementary Information

Supplementary Information.

## Data Availability

Chinese Center for Disease Control and Prevention has published the COVID-19 situation since Jan 16th. Everyone can obtain the daily confirmed COVID-19 cases from http://2019ncov.chinacdc.cn/2019-nCoV/. This research has been conducted using the confirmed COVID-19 cases from 22 January 2020 to 4 March.
