# Supplementary material for: Summary of the COVID-19 epidemic and estimating the effects of emergency responses in China
Source: Sci Rep. 2021 Jan 12;11:717. doi: 10.1038/s41598-020-80201-8 (PMC7803947; doi:10.1038/s41598-020-80201-8)
Supplement: Supplementary file 1 — Supplementary Information. [file 41598_2020_80201_MOESM1_ESM.pdf]

Supplementary Appendix - Summary of the COVID-19 epidemic and estimating the effects of emergency responses in China

Junwen Tao[1,2], Yue Ma[1,2], Caiying Luo[1], Jiaqi Huang[1], Tao Zhang[1], Fei Yin[1,*]

1: West China School of Public Health and West China Fourth Hospital, Sichuan University

2: These authors contributed equally to this article and share the first authorship

*: Corresponding author: Fei Yin, email: scupublichealth@163.com

In order to assess the impact of the emergency response implemented in each province, we fitted the growth curves at two different periods, using the emergency response time and the average incubation period of seven days as the cut-off point.

Supplementary Table S1 Emergency responses implemented date and two periods of fitting logistic growth model before and after the emergency responses

Area	Emergency response date	Time period one	Time period two
China	2020/1/24	2020/1/22-1/31	2020/2/1-3/4
Hubei	2020/1/24	2020/1/22-1/31	2020/2/1-3/4
China except for Hubei	2020/1/24	2020/1/22-1/31	2020/2/1-3/4
Sichuan	2020/1/24	2020/1/22-1/31	2020/2/1-3/4
Guangdong	2020/1/23	2020/1/22-1/30	2020/1/31-3/4
Beijing	2020/1/24	2020/1/22-1/31	2020/2/1-3/4
Shandong	2020/1/24	2020/1/22-1/31	2020/2/1-3/4
Chongqing	2020/1/24	2020/1/22-1/31	2020/2/1-3/4

Zhejiang	2020/1/23	2020/1/22-1/30	2020/1/31-3/4
Jiangxi	2020/1/24	2020/1/22-1/31	2020/2/1-3/4
Anhui	2020/1/24	2020/1/22-1/31	2020/2/1-3/4
Jiangsu	2020/1/25	2020/1/22-2/1	2020/2/2-3/4
Hunan	2020/1/24	2020/1/22-1/31	2020/2/1-3/4
Shanghai	2020/1/24	2020/1/22-1/31	2020/2/1-3/4
Henan	2020/1/25	2020/1/22-2/1	2020/2/2-3/4

The coefficients of the Logistic growth curve models before and after emergency response in China, Hubei province, and twelve high-risk provinces.

Supplementary Table S2 The coefficients of the logistic growth curve models before the emergency responses

Area	K_1[a]	N_0_1[b]	r_1[c]	R^2_1[d]
China	14650	158	0.565	0.971
Hubei	8495	63	0.614	0.967
China except for Hubei	6068	104	0.508	0.969
Sichuan	275	6	0.475	0.959
Guangdong	584	10	0.498	0.969
Beijing	180	7	0.443	0.950
Shandong	231	4	0.584	0.966

Chongqing	268	13	0.45	0.919
Zhejiang	853	6	0.603	0.999
Jiangxi	440	3	0.576	0.994
Anhui	425	8	0.469	0.972
Jiangsu	296	4	0.509	0.970
Hunan	443	5	0.625	0.966
Shanghai	223	4	0.44	0.984
Henan	632	11	0.468	0.953

a: K_1: The maximum cumulative confirmed cases before the emergency responses.

b: N_0_1: The cumulative confirmed cases at the initial time before the emergency responses.

c: r_1: The average increase rate of the cumulative confirmed cases before the emergency responses.

d: R^2_1: The coefficient of determination before the emergency responses.

Supplementary Table S3 The coefficients of the logistic growth curve models after the emergency responses

Area	K_2[a]	N_0_2[b]	r_2[c]	R^2_2[d]
China	66050	120	0.314	0.990
Hubei	58213	70	0.328	0.997
China except for Hubei	7649	25	0.343	0.963
Sichuan	304	2	0.279	0.948
Guangdong	820	3	0.37	0.966

Beijing	225	1	0.308	0.966
Shandong	568	10	0.179	0.925
Chongqing	311	2	0.313	0.966
Zhejiang	606	1	0.435	0.951
Jiangxi	604	1	0.397	0.969
Anhui	647	1	0.417	0.986
Jiangsu	361	0	0.393	0.968
Hunan	551	1	0.418	0.964
Shanghai	157	0	0.402	0.967
Henan	702	1	0.393	0.973

a: K_2: The maximum cumulative confirmed cases after the emergency responses.

b: N_0_2: The cumulative confirmed cases at the initial time after the emergency responses.

c: r_2: The average increase rate of the cumulative confirmed cases after the emergency responses.

d: R^2_2: The coefficient of determination after the emergency responses.
